# Yield of Clinical Screening for Hypertrophic Cardiomyopathy in Child First-Degree Relatives: Evidence for a Change in Paradigm

**DOI:** 10.1161/CIRCULATIONAHA.118.038846

**Published:** 2019-04-22

**Authors:** Gabrielle Norrish, Joanna Jager, Ella Field, Ellie Quinn, Hannah Fell, Emma Lord, Marcos N. Cicerchia, Juan Pablo Ochoa, Elena Cervi, Perry M. Elliott, Juan Pablo Kaski

**Affiliations:** 1 Centre for Inherited Cardiovascular Diseases, Great Ormond Street Hospital, London UK (G.N., J.J., E.F., H.F., E.L., E.C., J.P.K.).; 2 Institute of Cardiovascular Sciences, University College London, UK (G.N., J.J., P.M.E., J.P.K.).; 3 St. Bartholomew’s Centre for Inherited Cardiovascular Disease, St. Bartholomew’s Hospital, West Smithfield, London, UK (E.Q., P.M.E.).; 4 Health in Code S.L., Scientific Department, A Coruña, Spain (M.N.C., J.P.O.).; 5 Member of ERN GUARDHEART (European Reference Network for Rare and Complex Diseases of the Heart (P.M.E., J.P.K.).

**Keywords:** cardiomyopathies, child, death, sudden, genetics, mass screening

## Abstract

**Background:**

Hypertrophic cardiomyopathy (HCM) is a heritable myocardial disease with age-related penetrance. Current guidelines recommend clinical screening of relatives beginning at 10 years of age, but the clinical value of this approach has not been systematically evaluated.

**Methods:**

Anonymized clinical data were collected from children referred for family screening between 1994 and 2017 after diagnosis of HCM in a first-degree relative.

**Results:**

Of 1198 consecutive children (≤18 years of age) from 594 families who underwent serial evaluation (median, 3.5 years; interquartile range, 1.2–7), 32 individuals met diagnostic criteria at baseline (median maximal left ventricular wall thickness, 13 mm; interquartile range, 8-21 mm), and 25 additional patients developed HCM during follow-up. Median age at diagnosis was 10 years (interquartile range, 4–13 years); 44 (72%) were ≤12 years of age. Median age of affected patients at the last follow-up was 14 years (interquartile range, 9.5–18.2 years). A family history of childhood HCM was more common in those patients diagnosed with HCM (n=32 [56%] versus n=257 [23%]; *P*<0.001). Eighteen patients (32%) were started on medication for symptoms; 2 (4%) underwent a septal myectomy; 14 (25%) received an implantable cardioverter-defibrillator; 1 underwent cardiac transplantation; 2 had a resuscitated cardiac arrest; and 1 died after a cerebrovascular accident.

**Conclusions:**

Almost 5% of first-degree child relatives undergoing screening meet diagnostic criteria for HCM at first or subsequent evaluations, with the majority presenting as preadolescents; a diagnosis in a child first-degree relative is made in 8% of families screened. The phenotype of familial HCM in childhood is varied and includes severe disease, suggesting that clinical screening should begin at a younger age.

## Abstract 2

Supplemental Digital Content is available in the text.

**Clinical Perspective**

**What Is New?**

- A diagnosis of hypertrophic cardiomyopathy is made in almost 5% of first-degree child relatives from 8% of families.
- The majority of diagnoses (72%) are made in preadolescence.
- A diagnosis of hypertrophic cardiomyopathy was more likely in the context of a family history of childhood-onset disease

**What Are the Clinical Implications?**

- The phenotype of familial hypertrophic cardiomyopathy in childhood is varied and includes severe disease, suggesting that clinical screening should begin at a younger age.

**Editorial, see p 193**

Hypertrophic cardiomyopathy (HCM) is a heritable myocardial disease characterized by left ventricular (LV) hypertrophy (LVH) unexplained by abnormal loading conditions. It is rare in childhood, with an estimated annual incidence of 0.24 to 0.47 per 100 000^1–3^ and prevalence of 2.7 per 100 000.^1^ The cause of childhood HCM is heterogeneous and includes inborn errors of metabolism, malformation syndromes, and neuromuscular disease.^4,5^ However, the majority of disease in childhood is caused by mutations in cardiac sarcomere protein genes,^6,7^ which are inherited as autosomal dominant traits but exhibit variable and age-related penetrance.^8^ Previous studies have suggested that LVH in familial and sarcomeric HCM usually develops during adolescence,^4,5,9,10^ and current clinical practice guidelines^11,12^ recommend family screening for first-degree child relatives beginning at 10 years of age. However, the clinical value of this approach has not been systematically assessed. The aim of this study was to describe the yield of clinical screening for HCM in childhood and adolescent first-degree relatives in a large referral center population.

## Methods

The data, analytical methods, and study materials will not be made available to other researchers for purposes of reproducing the results or replicating the procedure.

### Patients

All patients ≤18 years of age referred between 1994 and 2017 to Great Ormond Street Hospital Center for Inherited Cardiovascular Diseases for family screening after a diagnosis of HCM in a first-degree relative were included in the study. Children referred for investigation of symptoms, with a previous diagnosis of HCM, or with a family history of nonsarcomeric HCM (including a malformation syndrome, neuromuscular disease, or inborn error of metabolism) were excluded.

### Clinical Evaluation

All patients underwent detailed evaluation at baseline and during follow-up (12–24 months during preadolescent years and 6–12 months during adolescent years) until they were transitioned to adult services (at 18 years of age) or the end of the study period. Anonymized, noninvasive clinical information was collected from baseline clinical evaluation, follow-up, and last clinical review, including demographics; symptoms; medical therapy; physical examination; family history; resting and ambulatory ECG; and 2D, Doppler, and color transthoracic echocardiography. A diagnosis of HCM was made if the LV wall thickness was >2 SDs above the body surface area–corrected population mean (*z* score >2) and could not be explained solely by abnormal loading conditions or in accordance with published criteria for familial disease.^11^

Echocardiographic measurements were made according to current guidelines.^13^ Specifically, end-diastolic LV wall thickness was measured by 2D echocardiography in the parasternal short-axis views in 4 places at the level of the mitral valve and papillary muscles (anterior and posterior septum, lateral and posterior wall) and in 2 places at apical level (anterior and posterior septum).^11^ Maximum LV wall thickness (MLVWT) was defined as the greatest thickness in any single segment. LV outflow tract (LVOT) obstruction was defined as an instantaneous peak Doppler LVOT pressure gradient ≥30 mm Hg at rest.^11^ A hemodynamically significant gradient was considered to be an instantaneous peak Doppler gradient ≥50 mm Hg.^14^ LV diastolic dysfunction was assessed to be present if 2 of 4 variables used to assess diastolic function were out of normal range for age and body surface area (annular E’ velocity, septal E’ velocity, average E/E’ ratio, left atrial volume).^15^ Twelve-lead ECGs for patients meeting diagnostic criteria for HCM were analyzed by 1 observer (G.N.) for the following: QRS axis, Sokolow-Lyon voltage criteria for LVH (V_1_+ RV5/6>35mV), abnormal Q waves, and repolarization abnormalities. Nonsustained ventricular tachycardia during ambulatory ECG monitoring was defined as ≥3 consecutive ventricular beats at a rate of ≥120 bpm with a duration of <30 seconds.^11^

### Genetic Testing

Sequencing methods varied according to year, panel, and the clinical laboratory conducting the testing. Before 2011, targeted testing of HCM genes (4–10 genes) was performed by direct Sanger sequencing. Next-generation sequencing (NGS) was available beginning in 2011. For the purpose of analysis, NGS panels were described as small (≤21 genes) or expanded (>21 genes). The genes included in panels varied depending on the year and clinical laboratory conducting the testing.

Data were collected from those families in whom genetic testing had been performed. Data included date of testing, genetic testing strategy, and variants identified.

The pathogenicity of all reported variants was reclassified by the authors according to the American College of Medical Genetic Classification.^16^

### Statistical Analysis

All statistical analyses were performed with STATA (Stata Statistical Software, release 14; StataCorp LP, College Station, TX). Body surface area was calculated from height and weight.^17^ MLVWT measurements are expressed in millimeters and as *z* scores relative to the distribution of measurements versus body surface area in normal children.^18^ Normally distributed continuous variables are described as mean±SD with 2-group comparisons conducted with the Student *t* test. Skewed data are described as median (interquartile range [IQR]) with 2-group comparisons performed with the Wilcoxon rank-sum test. To determine the association between relevant predictors, univariable analysis was performed with the χ^2^ test or Fisher exact test. A value of *P*<0.05 was accepted as significant for all analyses. Locally weighted scatterplot smoothing was performed for all line graphs.

This study was approved by Great Ormond Street Hospital/University College London Institute of Child Health Research and Development Office.

## Results

A total of 1198 consecutive pediatric first-degree relatives from 594 families were referred for clinical family screening over the study period. The number of patients evaluated per calendar year is shown in Figure I in the online-only Data Supplement. Mean±SD age at referral was 7.9±4.7 years (range, 0–18 years); 964 patients (80%) were ≤12 years of age at baseline evaluation, and 387 patients (32%) were transitioned to adult services by the end of the study period.

### Yield of Clinical Screening

**Figure 1.**
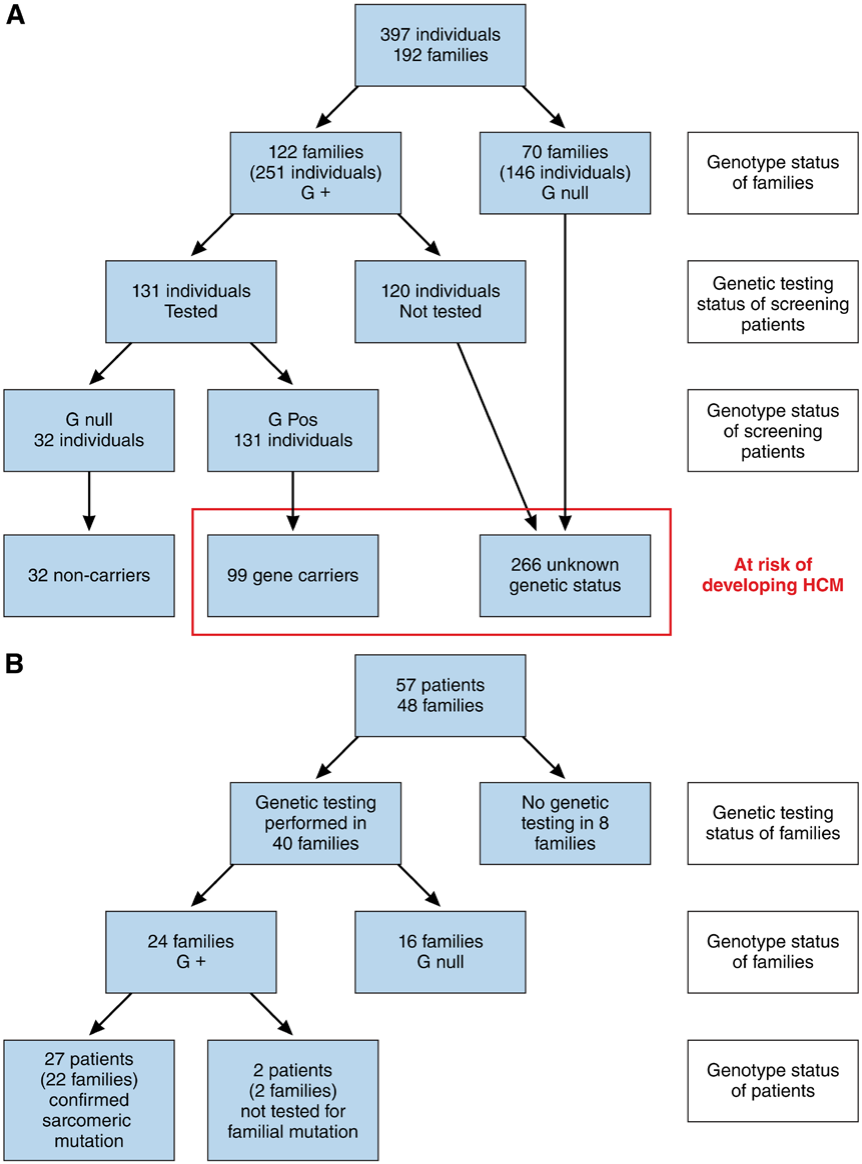
**Genetic testing in pediatric patients with hypertrophic cardiomyopathy (HCM).** **A**, Genetic testing in patients referred for clinical screening. **B**, Genetic testing in patients diagnosed with HCM through family screening. G+ indicates genetically tested and pathogenic sarcomeric mutation identified; G null, genetically tested and no pathogenic sarcomeric mutation identified; and Pos, positive.

Over a median follow-up of 3.5 years (IQR, 1.2–7 years), 57 patients (4.7%) from 48 unrelated families were diagnosed with childhood HCM. A diagnosis in a first-degree child relative was made in 8.1% of families screened. The yield of clinical screening did not differ by era of screening (Table 1). Age at diagnosis was <1 year in 6 patients (11%), 1 to 6 years in 15 (26%), 7 to 12 years in 20 (35%), and >12 years in 16 (28%; Figure 1B). Median age at diagnosis was 10 years (IQR, 4–13 years). Thirty-two individuals met diagnostic criteria at baseline, and 25 additional patients developed HCM during follow-up. The age at baseline evaluation did not differ between these groups (baseline diagnosis [n=32]: median age, 5 years [IQR, 1–11.5 years]; diagnosis during follow-up [n=25]: median age, 5 years [IQR, 4–9 years]; *P*=0.872); however, those diagnosed during follow-up were older at the time of diagnosis (median, 12 years [IQR, 9–14 years] compared with 6 years [IQR, 1–11.5 years]; *P*=0.02). Table 1 compares the demographics of those with and without a diagnosis of HCM. Patients with a childhood diagnosis were more likely to have a family history of childhood HCM (n=32 [56%] versus n=257 [23%]; *P*<0.001). Of this group, 148 (12.3%) had an affected pediatric sibling as one of their first-degree relatives.

**Table 1.**
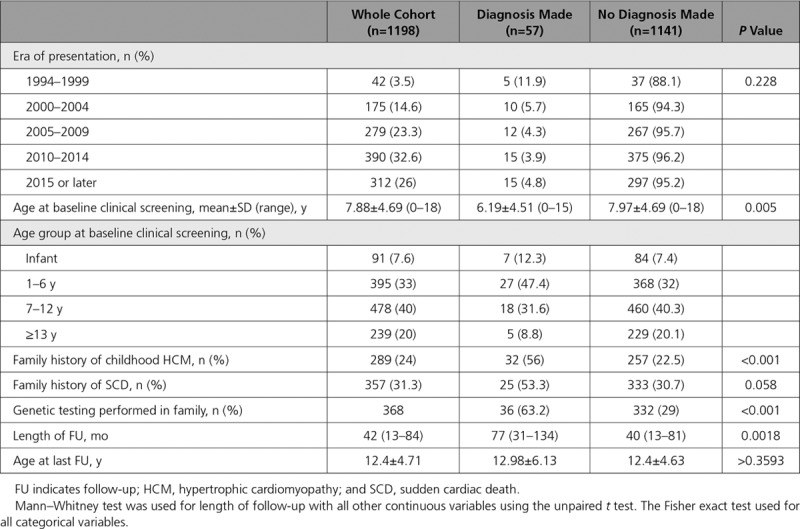
Baseline Demographics in Patients With and Without a Diagnosis of HCM

### Genetic Testing

The genetic testing strategy for the whole cohort is shown in Figure 1A. In brief, genetic testing was performed in 192 families (32%), with a pathogenic or likely pathogenic sarcomeric variant identified in 122 (64%). Of variants previously classified as pathogenic/likely pathogenic, after ACMG reclassification, 87 variants remained pathogenic/likely pathogenic and 24 were reclassified (variant of unknown significance, n=22; benign variant, n=2). Seven variants previously classified as variants of unknown significance were reclassified to pathogenic/likely pathogenic (Table I in the online-only Data Supplement). The genetic testing strategy in the 57 pediatric patients diagnosed through family screening is shown in Figure 1B. In brief, genetic testing for sarcomeric mutations was performed in 39 individuals (68%; 40 families, 83%), identifying a pathogenic sarcomeric variant in 27 (69%) individuals: *MYH7*, n=18; *MYBPC3*, n=7; *TPM1*, n=1; *MYBPC3*+*TNNT2*, n=1). Twenty-two patients (39%) underwent predictive testing for a familial sarcomeric gene variant, and 17 (30%) underwent gene panel testing. The sequencing method and number of genes tested in the genetic index case were as follows: Sanger sequencing, 9 (22.5%); small NGS panel, 13 (32.5%); expanded NGS panel, 9 (22.5%); and unknown, 9 (22.5%). Sixteen families underwent genetic testing with no pathogenic variant identified: Sanger sequencing, n=6; small NGS panel, n=3; extended NGS panel, n=6; and unknown panel, n=1. The genetic testing strategy by era is shown in Table II in the online-only Data Supplement. The yield of genetic testing by year of presentation is shown in Figure II in the online-only Data Supplement. Median age at diagnosis for sarcomeric mutation carriers was 6 years (IQR, 3.75–10 years); 21 (78%) were <10 years of age.

### Phenotype at Baseline of Patients Meeting Diagnostic Criteria for HCM

Table 2 describes the baseline clinical features of the 57 patients diagnosed with HCM through clinical screening. Of 32 patients meeting diagnostic criteria for HCM at baseline, 4 (13%) reported previous cardiac symptoms (chest pain [n=2], dyspnea [n=2]). Twenty-eight (88%) had asymmetrical septal hypertrophy with a median MLVWT of 13 mm (IQR, 8–21 mm) and mean *z* score of 8.9 (±5.4); no patient had an MLVWT ≥30 mm. Three patients (6%) had resting LVOT obstruction. Twenty-eight patients (88%) had abnormalities on a resting 12-lead ECG.

**Table 2.**
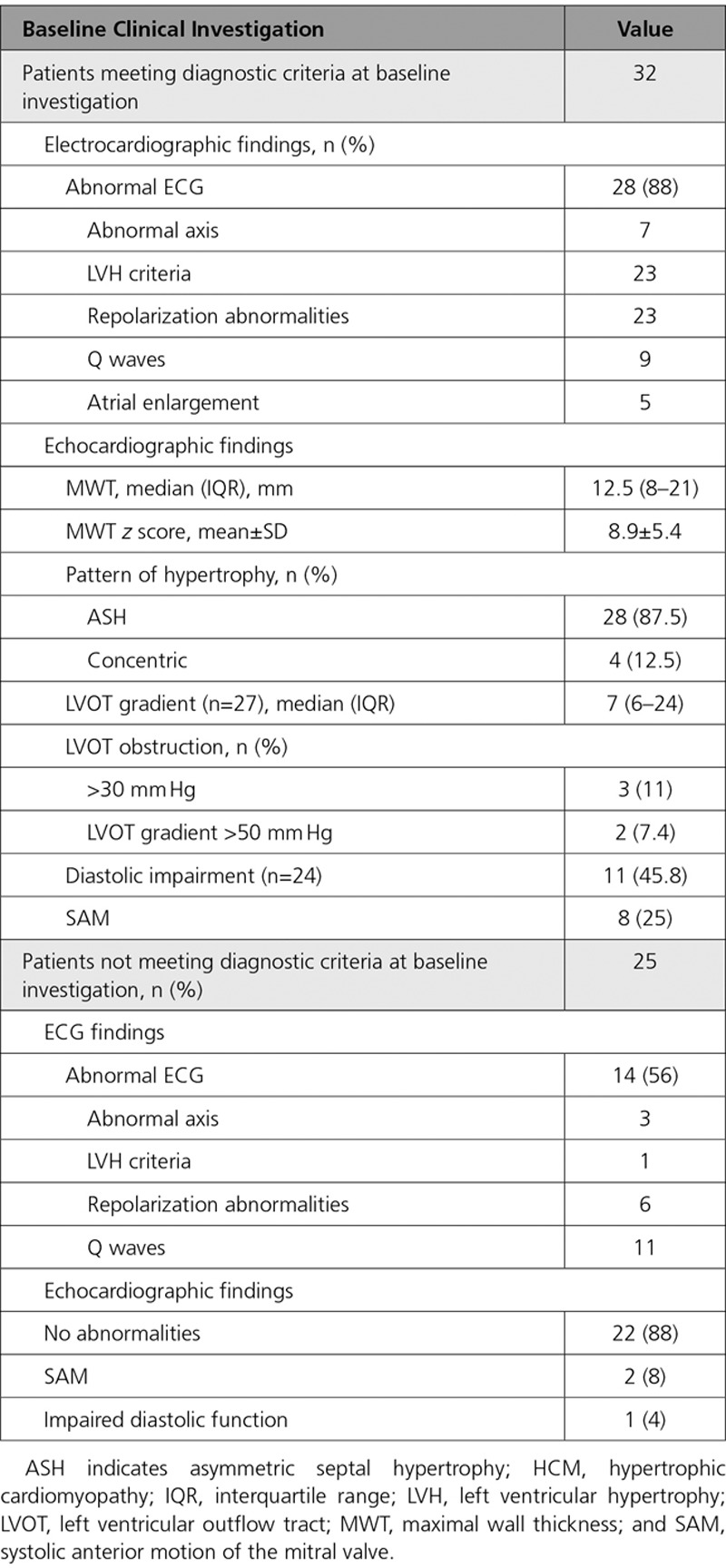
Baseline Investigations for Patients Diagnosed With HCM Through Family Screening

Of 25 patients not meeting diagnostic criteria at baseline assessment but who developed HCM during follow-up in childhood, 14 (56%) had abnormalities on a resting 12-lead ECG, and 3 had nondiagnostic echocardiographic abnormalities (impaired diastolic function, n=1; incomplete systolic motion of the mitral valve, n=2) at baseline evaluation.

### Disease Progression in Patients Meeting Diagnostic Criteria for HCM

**Figure 2.**
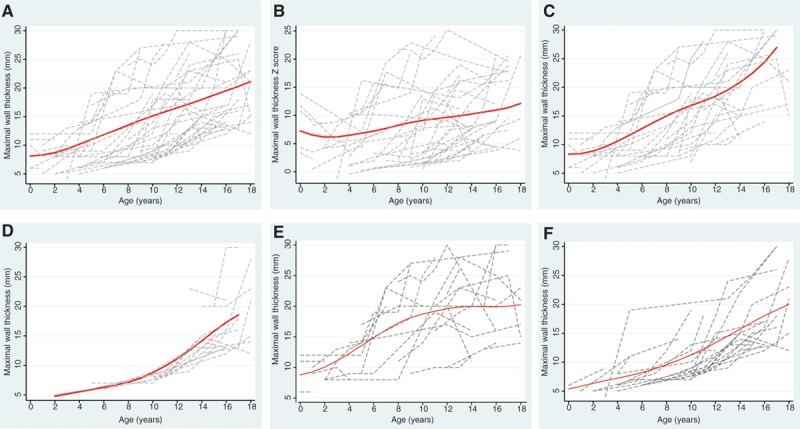
**Progression of left ventricular hypertrophy during childhood. A**, Change in absolute maximal left ventricular wall thickness (MLVWT) during childhood in those patients diagnosed through clinical screening (n=48). **B**, Change in MLVWT *z* score during childhood in those patients diagnosed through clinical screening (n=48). **C**, Change in absolute MLVWT during childhood in those patients diagnosed in preadolescence (≤12 years of age; n=32). **D**, Change in absolute MLVWT during childhood in those patients diagnosed in adolescence (≥13 years of age; n=16). **E**, Change in absolute MLVWT during childhood in those patients diagnosed at baseline evaluation (n=32). **F**, Change in absolute MLVWT during childhood in those patients diagnosed during follow-up (n=25). Connected dash line represents serial measurements from a single patient. Red line represents locally weighted scatterplot smoothing.

Patients with a diagnosis of HCM were followed up for a median of 7.3 years (IQR, 2.7–12.8 years). Nine patients (16%) had <1 year of follow-up. For 48 patients in whom serial echocardiographic measurements were available, MLVWT increased at a median rate of 0.8 mm/y (range, −0.7 to 3.9 mm/y; IQR, 0.4–1.6 mm/y; Figure 2). At the last clinical follow-up, 52 patients (91%) had asymmetrical septal hypertrophy with a median MLVWT of 17 mm (IQR, 12.5–24.5 mm). Five patients had a maximal wall thickness ≥30 mm. Median LVOT gradient was 9 (IQR, 6–13); 2 patients had LVOT obstruction at rest. Only 3 patients (5%) had no abnormalities on the 12-lead ECG.

### Clinical Outcome of Patients Meeting Diagnostic Criteria for HCM

During clinical follow-up, 17 patients (30%) reported cardiac symptoms (palpitations, n=6; dyspnea, n=4; chest pain, n=5;and presyncope/syncope, n=3), and 18 (32%) were started on medications. Indications for starting medical therapy are described in Table 3. Two patients underwent a myectomy, and 4 had an electrophysiology study. Fourteen patients (25%) received an implantable cardioverter-defibrillator (ICD): 2 for secondary prevention after a resuscitated cardiac arrest at 14 and 25 years of age and 12 for primary prevention of malignant arrhythmias (Table 3). Over a median follow-up of 5.7 years (IQR, 2.1–6.7 years), 1 patient (26 years of age) received multiple appropriate therapies; 1 patient (20 years of age) received inappropriate ICD therapy and was found to have a lead fracture; 1 patient (22 years of age) developed infective endocarditis with ICD lead vegetations; and 2 patients required ICD lead replacement as a result of somatic growth. Fifty-eight patients (98%) were alive and well at the last clinical follow-up with a median age of 14 years (IQR, 9.5–18.2 years); 16 patients (28%) were >18 years of age. One patient died as a result of a cerebrovascular accident at 24 years of age. One patient progressed to end-stage HCM necessitating cardiac transplantation at 15 years of age. In this family, mitochondrial disease was initially suspected as the phenotype and included preexcitation on 12-lead resting ECG, recurrent supraventricular and ventricular arrhythmias, retinitis pigmentosa, and early progression to end-stage disease in the patient and her mother. However, genetic testing on an expanded NGS panel and metabolic investigations, including a muscle biopsy, did not identify an underlying cause.

**Table 3.**
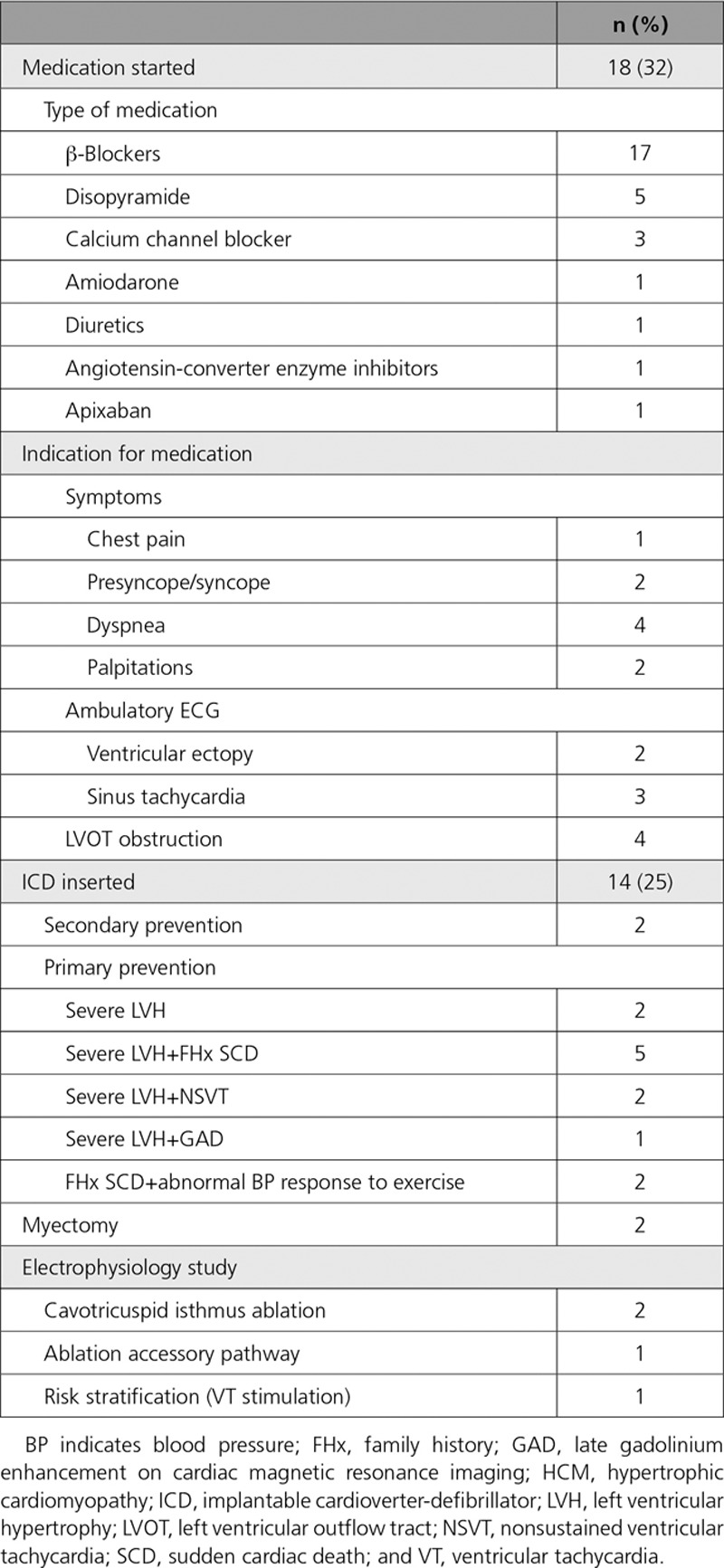
Management of Patients With a Diagnosis of HCM

## Discussion

This study is, to the best of our knowledge, the first to describe the yield of clinical screening for HCM in first-degree relatives in a large unselected consecutive childhood cohort. The results suggest that clinical screening for HCM in first-degree relatives should be considered earlier than recommended by current international guidelines.

### Clinical Yield of Screening During Childhood

Cascade family screening to identify asymptomatic individuals is widely accepted as an important part of HCM management, but there are no data on the clinical yield of screening in adult or pediatric HCM relatives. The present study demonstrates that clinical screening for HCM in childhood results in a diagnosis in almost 1/10th of families, with high variability in the age at which a phenotype develops. In the absence of a malignant family history, symptoms, or involvement in competitive sports, current guidelines do not recommend screening during childhood before 10 years of age. These recommendations are derived largely from expert opinion based on reports that development of a phenotype is rare during childhood, progression of LVH is most commonly seen during adolescence,^9^ and adverse events rarely occur in childhood.^4,5^ However, we have shown that, in most cases when a diagnosis is made in childhood, it occurs in preadolescence. Furthermore, although patients with a diagnosis made through screening in childhood were more likely to have a family history of childhood disease, this accounts for only half of patients with early-onset disease. The results of this study represent a paradigm shift and support the notion that, if it is accepted that screening is important, consideration should be given to beginning screening for familial disease at a younger age.

### Importance of an Early Diagnosis

Early diagnosis of HCM through family screening enables appropriate treatment to be instigated promptly. Although most patients undergoing family screening are asymptomatic, symptoms attributable to HCM are often nonspecific, meaning that delays in management are common because cardiac investigations may not be initially considered. Early diagnosis also facilitates surveillance for disease complications such as malignant arrhythmias, which may occur even in asymptomatic individuals. In this cohort, diagnosis resulted in a change in management (medication for symptoms, ICD implantation, or myectomy) for more than one-third of patients. Of note, this included 1 patient who received appropriate ICD therapies having undergone primary prevention ICD implantation. In contrast, 2 patients without an ICD had an out-of-hospital resuscitated cardiac arrest, highlighting the challenges of risk stratification in childhood HCM. Recent animal data have shown that novel compounds may have a role in preventing disease expression in HCM.^19^ In the future, early detection of these patients through family screening will be important to identify a group of patients likely to benefit from such therapies.

### Progression of Familial HCM During Childhood

Our understanding of the progression of familial disease in childhood remains incomplete. Maron et al^9^ described the progression of LVH in a small childhood cohort (39 patients) and found that increases in wall thickness were seen more frequently in adolescence. However, this study contained a small number of preadolescent patients (n=10), of whom 40% had preexisting LVH. In comparison, most patients with childhood disease in our cohort were first evaluated at <12 years of age, and the majority developed LVH in preadolescence. After diagnosis, increases in the absolute and body surface area–corrected maximal wall thickness occurred throughout childhood. Progression of LVH in patients diagnosed later in childhood reflected that previously described, with increases in wall thickness occurring during adolescence. This suggests that earlier screening identifies 2 distinct groups: a substantial minority who have evidence of HCM in early childhood with a natural history similar to that previously described but shifted to the left, and a second, larger group in whom the disease may not develop until adulthood. Several patients in our cohort reached a peak maximal wall thickness during childhood, with regression of hypertrophy in early adulthood, and 1 patient developed end-stage disease requiring a heart transplantation during childhood. Progression to a dilated, hypokinetic “burnt-out” phase is exceedingly rare in nonmetabolic childhood HCM,^20^ and extensive genetic testing failed to identify a sarcomeric mutation in this patient, suggesting an alternative pathogenesis.

### Genetic Testing

Although the impact of genetic testing was not the focus of this study, our findings raise the important question of whether predictive genetic testing may be a more cost-effective way to screen pediatric relatives of HCM than clinical screening.^21^ In routine clinical practice, however, the family genotype may not always be known. In this study, genetic testing over the study period was not systematic and was performed primarily on a research basis,^7,22^ explaining the relatively low proportion of genotyped families in this cohort. Nevertheless, in those children with a diagnosis of HCM, more than two-thirds (68%) have undergone genetic testing, identifying a pathogenic sarcomeric variant in 69%. The increasing use of predictive testing since 2016 reflects the more widespread availability of genetic testing in both pediatric and adult cardiomyopathy services. This study did not attempt to investigate the penetrance of sarcomeric mutations during childhood but does provide further evidence that sarcomeric disease can present in younger children.^6,7^

### Limitations

As a result of the retrospective nature of the study and the fact that many families were seen before the availability of widespread genetic testing, the clinical yield of screening childhood first-degree relatives reported in this study is likely to be an underestimate of the true penetrance of childhood sarcomeric disease because the cohort necessarily contains both genotype-positive and -negative individuals. This reflects real-world clinical practice in which the genotype status of a child is often unknown. This study included data only on children referred for screening after a diagnosis of HCM in a first-degree relative, and the findings may not be applicable to the general pediatric HCM population. In particular, the data on disease progression relate to patients with childhood familial HCM diagnosed through clinical screening and therefore may not be generalizable to those presenting with symptoms or with HCM diagnosed as an incidental finding. Further work to explore the age-related, gene-related, and mutation-specific penetrance of sarcomeric disease in childhood is needed.

### Conclusions

In a large, unselected, consecutive childhood cohort, almost 5% of first-degree child relatives undergoing screening meet diagnostic criteria for HCM at first or subsequent evaluations, with the majority presenting as preadolescents. Furthermore, a diagnosis of HCM in at least 1 pediatric first-degree relative is made in 8% of families screened. A diagnosis of HCM was more likely in the context of a family history of childhood-onset disease. The phenotype of familial HCM in childhood is varied and includes severe disease, suggesting that clinical screening should begin at a younger age than currently recommended.

## Acknowledgments

University College London and St. Bartholomew’s Hospital (London EC1A 7BE, UK) and Great Ormond Street Hospital for children (London, WC1N 4JH, UK) are members of ERN GUARD-HEART (European Reference Network for Rare and Complex Diseases of the Heart; http://guardheart.ern-net.eu).

## Sources of Funding

This work was supported by the British Heart Foundation (grant FS/16/72/32270) to Drs Norrish and Kaski. E. Field and Kaski are supported by Max’s Foundation and Great Ormond Street Hospital Children’s Charity. This work is (partly) funded by the National Institute for Health Research Great Ormond Street Hospital Biomedical Research Centre. The views expressed are those of the authors and not necessarily those of the National Health Service, the National Institute for Health Research, or the Department of Health. Some of this work was undertaken at University College London (United Kingdom) and St. Bartholomew’s Hospital (London, UK), which received a portion of funding from the UK Department of Health’s National Institute for Health Research Biomedical Research Centres funding scheme.

## Disclosures

Drs Norrish and Kaski are supported by the British Heart Foundation (grant FS/16/72/32270). The other authors report no conflicts.

## Supplementary Material
