# Supplementary material for: Yield of Clinical Screening for Hypertrophic Cardiomyopathy in Child First-Degree Relatives: Evidence for a Change in Paradigm
Source: Circulation. 2019 Apr 22;140(3):184–92. doi: 10.1161/CIRCULATIONAHA.118.038846 (PMC6636798; doi:10.1161/CIRCULATIONAHA.118.038846)
Supplement: Supplementary file 1 [file cir-140-184-s001.pdf]

SUPPLEMENTAL MATERIAL

Supplementary Figure 1: Number of patients undergoing clinical screening by year

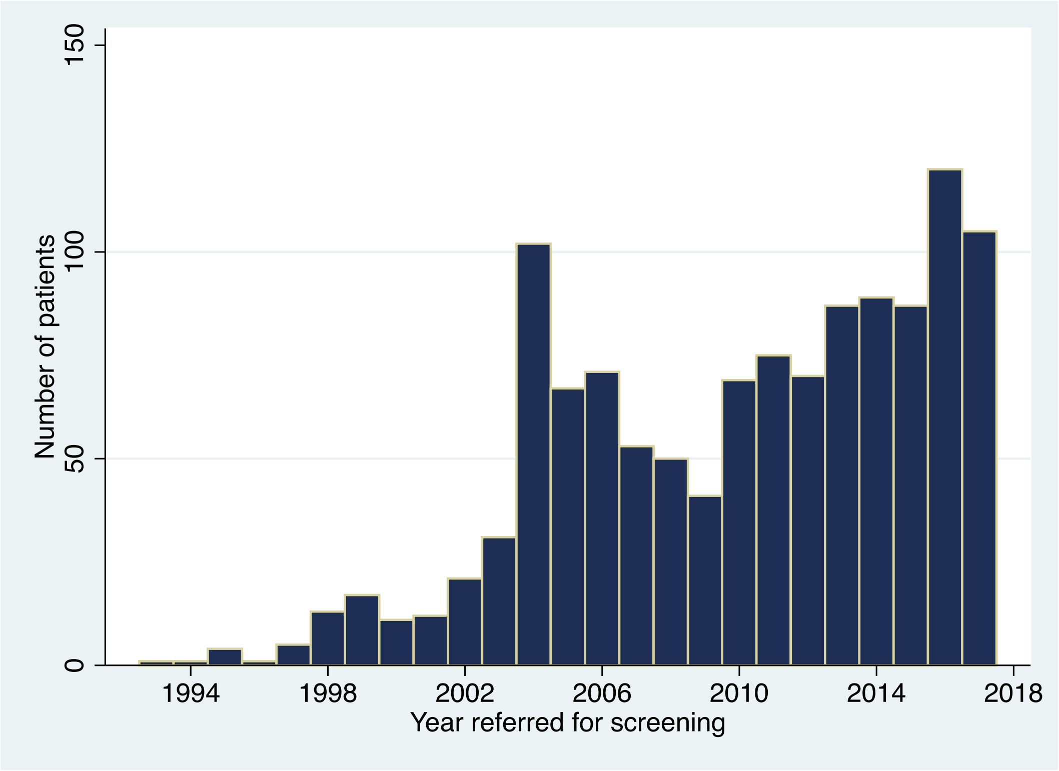

Supplementary figure 2: Result of genetic testing in patients with a diagnosis of HCM by era of diagnosis

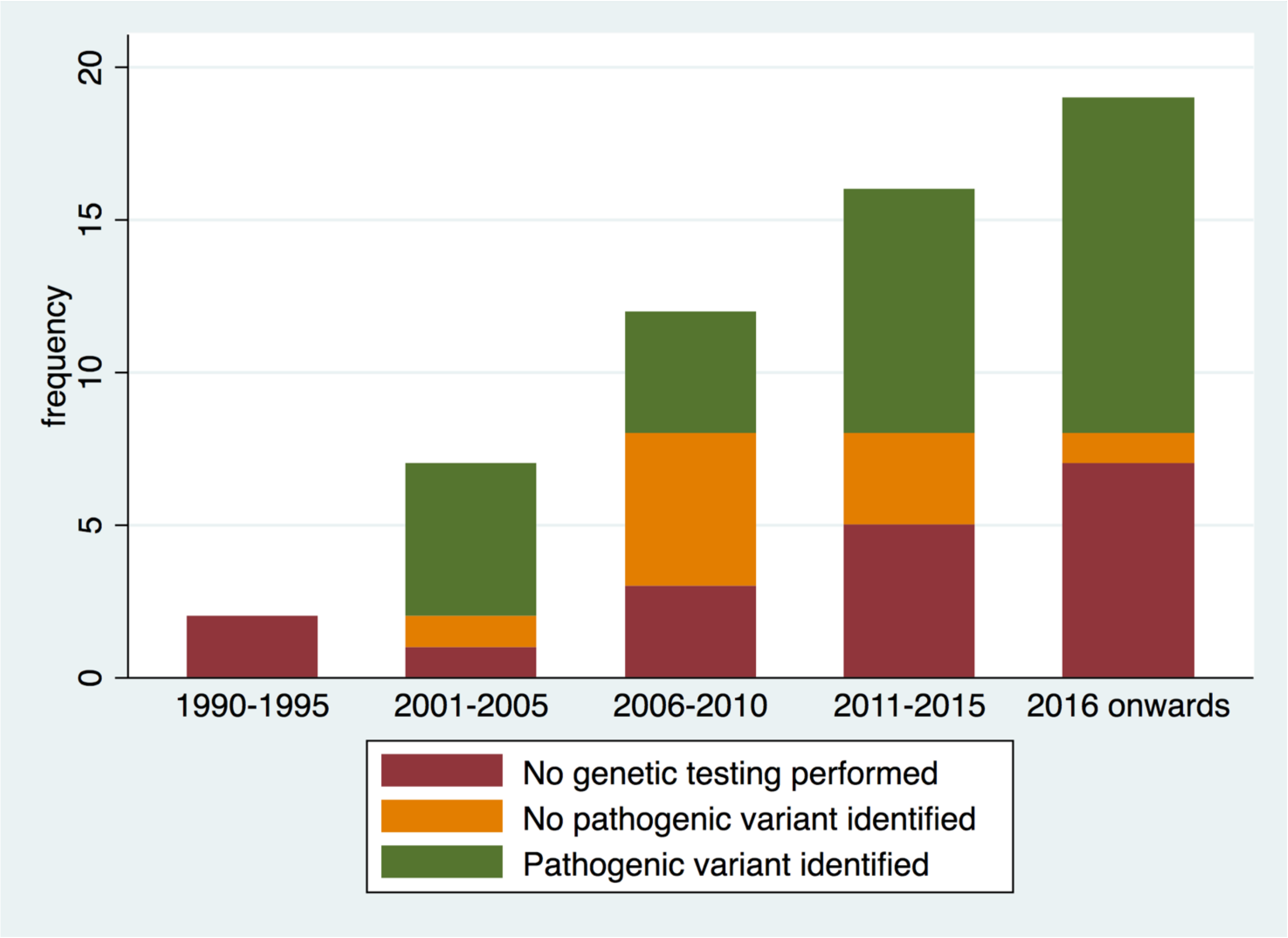

Supplementary table 1: Re-classification of reported variants according to ACMG classification guidance.

Gene	Protein change	Nucleotide change	Previous pathogenicity classification	ACMG classification
MYBPC3	Arg943*	c.2827C>T	**PATHOGENIC**	**PATHOGENIC**
TPM1	Glu192Lys	c.574G>A	**PATHOGENIC**	**LIKELY PATHOGENIC**
MYBPC3		c.3752_3753delAT	**PATHOGENIC**	**VUS**
MYH7	Glu374Val	g.23899001T>A	**PATHOGENIC**	**VUS**
MYBPC3	Arg495Gln	c.1484G>A	**PATHOGENIC**	**PATHOGENIC**
MYBPC3		c.927-9G>A	**PATHOGENIC**	**PATHOGENIC**
PRKAG2	Asn247Ile		**PATHOGENIC**	**VUS**
MYH7	Arg723Gly	c.2167C>G	**PATHOGENIC**	**PATHOGENIC**
MYL2	Gly87Ala	c.260G>C	**PATHOGENIC**	**VUS**
TNNT2	Glu163del	c.487_489delGAG	**PATHOGENIC**	**VUS**
MYBPC3		1928-2A>G	**PATHOGENIC**	**PATHOGENIC**
MYBPC3	Trp792Valfs*41	c.2373_2374insG	**PATHOGENIC**	**PATHOGENIC**
TNNI3	Asp196Asn	c.586G>A	**PATHOGENIC**	**PATHOGENIC**
TNNI3 +DES	Lys206Gln Leu470PhE	c.616A>C c.1408C>T	**PATHOGENIC VUS**	**PATHOGENIC VUS**
TPM1	Glu192Lys	c.574G>A	**PATHOGENIC**	**LIKELY PATHOGENIC**
MYH7	Phe247Leu	c.739T>C	**PATHOGENIC**	**VUS**
MYH7 + FHOD3	Asn696Ser Arg638Trp	c.2087A>G Arg638Trp	**PATHOGENIC VUS**	**LIKELY PATHOGENIC VUS**
MYH7	Glu374Lys	g.23899001T>A	**PATHOGENIC**	**VUS**
TNNT2	Arg92Gln	c.275G>A	**PATHOGENIC**	**PATHOGENIC**
MYBPC3		c. 3491-2A>T	**PATHOGENIC**	**PATHOGENIC**
MYBPC3	Lys505*	c.1513A>T	**PATHOGENIC**	**PATHOGENIC**
MYBPC3	Glu258Lys	c.772G>A	**PATHOGENIC**	**PATHOGENIC**
MYH7	Lys847Glu	c.2539A>G	**PATHOGENIC**	**PATHOGENIC**
MYBPC3 + PLN	Glu542Gln Ile47fs	c. 1624G>C c.138dupT	**PATHOGENIC VUS**	**PATHOGENIC VUS**
MYBPC3	His390Metfs16	c.1168delC	**PATHOGENIC**	**PATHOGENIC**
MYH7	Met982Thr	c.2945T>C	**PATHOGENIC**	**LIKELY BENIGN**
MYBPC3	Arg1138His	c.3413G>A	**PATHOGENIC**	**LIKELY BENIGN**
MYH7	Arg721Lys	c.2162G>A	**PATHOGENIC**	**LIKELY PATHOGENIC**
MYH7	Thr449Asn	c.1346C>A	**PATHOGENIC**	**VUS**
MYBPC3 X2	Arg802Trp Ser858Asn	c.2573G>A	**PATHOGENIC**	**VUS VUS**

MYBPC3		c.1224-19G>A	**PATHOGENIC**	**PATHOGENIC**
MYH7	Gly741Trp	c.2221G>T	**PATHOGENIC**	**PATHOGENIC**
TNNT2	Asp86Tyr	c.256G>T	**PATHOGENIC**	**LIKELY PATHOGENIC**
TNNT2 +<br>MYH7	Arg278Cys<br>Asp239Asn	c.832C>T<br>c.715G>A	**PATHOGENIC**	**PATHOGENIC**<br>**VUS**
TNNT2	Arg92Leu	c.832C>T	**PATHOGENIC**	**PATHOGENIC**
MYH7	Arg453Cys	c.1357C>T	**PATHOGENIC**	**PATHOGENIC**
MYH7	Glu931del	2791_2793delGAG	**PATHOGENIC**	**VUS**
MYH7	Ala797Thr	c.2389G>A	**PATHOGENIC**	**PATHOGENIC**
MYBPC3 +<br>TNNT2	Arg502Trp	c.1504C>T<br>(c.341C>T)	**PATHOGENIC**	**PATHOGENIC**
MYBPC3 +<br>FLNC	 Leu2421His	c.927-2A>G<br>c.7262T>A	**PATHOGENIC**<br>**VUS**	**PATHOGENIC**<br>**VUS**
MYH7	Arg442Cys	c.1324C>T	**PATHOGENIC**	**PATHOGENIC**
TNNT2	Arg148Gln	c.443A>G	**PATHOGENIC**	**VUS**
MYH7	Glu927Lys	c.2779G>A	**PATHOGENIC**	**PATHOGENIC**
MYBPC3	Ser871Glnfs*13	c.2610_2611insC	**PATHOGENIC**	**PATHOGENIC**
MYH7	Leu908Val	c.2722C>G	**PATHOGENIC**	**PATHOGENIC**
MYBPC3	Arg597Gln	c.1790G>A	**PATHOGENIC**	**PATHOGENIC**
MYBPC3	Arg502Trp	c.1504C>T	**PATHOGENIC**	**PATHOGENIC**
MYH7	Arg663His	c.1988G>A	**PATHOGENIC**	**PATHOGENIC**
MYH7	Leu889His	c.2666T>A	**PATHOGENIC**	**LIKELY PATHOGENIC**
MYH7	Ala335Thr	c.1003G>A	**PATHOGENIC**	**VUS**
MYBPC3 +<br>TNNT2	Arg495Gln<br>Arg285Cys	c.1484G>A<br>c.853G>A	**PATHOGENIC**<br>**VUS**	**PATHOGENIC**<br>**VUS**
MYH7 +<br>DES	Arg453Pro<br>Thr76Profs*22	c.1358G>C<br>c.226delA	**PATHOGENIC**<br>**VUS**	**PATHOGENIC**<br>**VUS**
MYH7	Asn817Lys	c.2451C>A	**PATHOGENIC**	**VUS**
MYBPC3	Glu1085Glyfs*104	c.3254delA	**PATHOGENIC**	**PATHOGENIC**
MYH7	Arg453His	c.1358G>A	**PATHOGENIC**	**PATHOGENIC**
MYH7	Glu958Lys	c.2872G>A	**PATHOGENIC**	**VUS**
MYBPC3	Ser871Alafs*8	c.2604_2605delTCinsA	**PATHOGENIC**	**PATHOGENIC**
MYBPC3	Pro699Glnfs*55	c2093delC	**PATHOGENIC**	**PATHOGENIC**
PRKAG2	Asn488Ile	c.1463A>T	**PATHOGENIC**	**VUS**
MYH7	Gly741Arg	c.2221G>C	**PATHOGENIC**	**PATHOGENIC**
MYBPC3	Val758Ala	c.2273C>T	**PATHOGENIC**	**LIKELY PATHOGENIC**
TNNT2	Arg92Trp	c.274C>T	**PATHOGENIC**	**PATHOGENIC**
TNNT2	Arg278Cys	c.832C>T	**PATHOGENIC**	**PATHOGENIC**
MYBPC3	Arg502Gln	c.1505G>A	**PATHOGENIC**	**PATHOGENIC**
MYH7	Glu965Lys	c.2893G>A	**PATHOGENIC**	**LIKELY PATHOGENIC**
MYBPC3 +<br>MYBPC3	Cys1266Tyr<br>Gly5Arg	c.3797G>A<br>c.13G>C	**PATHOGENIC**<br>**VUS**	**VUS**<br>**LIKELY BENIGN**
MYBPC3	Pro1243Arg	c.3728C>G	**PATHOGENIC**	**VUS**
MYBPC3	Tyr333*	c.999C>G	**PATHOGENIC**	**PATHOGENIC**
TNNI3	Arg162Gln	c.485G>A	**PATHOGENIC**	**PATHOGENIC**
MYBPC3	Arg495Gly	c.1483C>G	**PATHOGENIC**	**PATHOGENIC**
MYBPC3		C.927-9G>A	**PATHOGENIC**	**PATHOGENIC**

MYH7	Glu924Lys	c.2770G>A	**PATHOGENIC**	**PATHOGENIC**
MYH7	Tyr624Asn	c.1870T>A	**PATHOGENIC**	**VUS**
MYBPC3		1457+5G>C	**PATHOGENIC**	**PATHOGENIC**
MYBPC3 + FHOD3	Arg502Trp Arg637Gln	c.1504C>T	**PATHOGENIC VUS**	**PATHOGENIC VUS**
MYH7	Arg403Leu	1208G>T	**PATHOGENIC**	**PATHOGENIC**
MYH7	Arg403Gln	c.1208G>A	**PATHOGENIC**	**PATHOGENIC**
MYH7	Met515Thr	c.1544T>C	**PATHOGENIC**	**LIKELY PATHOGENIC**
MYBPC3	Gln969*	c.2905C>T	**PATHOGENIC**	**PATHOGENIC**
TNNT2	Arg92Trp	c.274C>T	**PATHOGENIC**	**PATHOGENIC**
MYBPC3		c.927-2A>G	**PATHOGENIC**	**PATHOGENIC**
ACTC1	Tyr296Asn		**PATHOGENIC**	**VUS**
MYBPC3	Tyr1251*	c.886T>A	**PATHOGENIC**	**PATHOGENIC**
MYBPC3		3190+5G>A	**PATHOGENIC**	**PATHOGENIC**
MIT1	m.4300A>G		**PATHOGENIC**	**VUS**
MYBPC3	Lys685Argfs	c.2054_2067+11del25	**PATHOGENIC**	**PATHOGENIC**
MYBPC3	Asp770Asn	c.2308G>A	**PATHOGENIC**	**PATHOGENIC**
MYBPC3 + MYH7	Arg502Trp Lys1459Asn	c.1504C>T c.4377G>T	**PATHOGENIC VUS**	**PATHOGENIC LIKELY BENIGN**
MYH7	(Asp900Glu)	c.2700T>A,	**PATHOGENIC**	**VUS**
MYH7	Arg719Gln	c.2156 G>A	**PATHOGENIC**	**PATHOGENIC**
TNNT2	Ile79Asn	c.236T>A	**PATHOGENIC**	**VUS**
MYBPC3		2326-36Del	**PATHOGENIC**	**PATHOGENIC**
MYBPC3	Trp1098*	c.3294G>A	**PATHOGENIC**	**PATHOGENIC**
MYBPC3	Pro960Leu		**PATHOGENIC**	**VUS**
MYH7	Val406Met	c.1216G>A	**PATHOGENIC**	**LIKELY PATHOGENIC**
MYBPC3+ MYBPC3 + CSRP3	Glu165Asp Thr84Met	3330+5G>A c.495G>C c.251C>T	**PATHOGENIC VUS VUS**	**PATHOGENIC VUS VUS**
MYBPC3+ MYH7	Asp610Asn Arg787His	c.1828G>A c.2360G>A	**PATHOGENIC PATHOGENIC**	**LIKELY PATHOGENIC LIKELY PATHOGENIC**
TNNI3	Arg162Glu	c.485G>A	**PATHOGENIC**	**PATHOGENIC**
MYBPC3	Pro453Cysfs*21	c.1357_1358delCC	**PATHOGENIC**	**PATHOGENIC**
MYBPC3		c.927-2A>G	**PATHOGENIC**	**PATHOGENIC**
MYBPC3	Asp1076Valfs*6	c.3226_3227insT	**PATHOGENIC**	**PATHOGENIC**
MYH7	Arg723Cys	c.2167C>T	**PATHOGENIC**	**PATHOGENIC**
MYH7	Ala355Ser	c.1063G>T	**PATHOGENIC**	**PATHOGENIC**
MYBPC3		c.1624+4A>T	**PATHOGENIC**	**PATHOGENIC**
MYH7	Ala463Asp	c.1388C>A	**PATHOGENIC**	**LIKELY PATHOGENIC**
MYBPC3 + MYL3	Tyr373* Ala57Asp	c.170C>A	**PATHOGENIC**	**PATHOGENIC LIKELY PATHOGENIC**
MYBPC3		c.1457+5G>C	**PATHOGENIC**	**PATHOGENIC**
MYH7	Glu256Glu	c.767G>A	**PATHOGENIC**	**PATHOGENIC**
TNNT2	Glul83Lys	c.277G>A	**PATHOGENIC**	**PATHOGENIC**
MYH7	Val606Leu	c.1816G>T	**PATHOGENIC**	**LIKELY PATHOGENIC**

MYH7	Asp906Gly	c.2717A>G	**VUS**	**PATHOGENIC**
MYBPC3	Lys505del	c.1513_1515delAAG	**VUS**	**PATHOGENIC**
MYBPC3 + MYH7	Asp610Asn Arg787His	c.1828G>A c.2360G>A	**VUS**	**LIKELY PATHOGENIC LIKELY PATHOGENIC**
MYH7	Glu1293Lys	c.3877G>A	**VUS**	**LIKELY PATHOGENIC**
TNNT2	Glu83Lys	c.277G>A	**VUS**	**PATHOGENIC**
TPM1	Ser252Thr	c.755G>C	**VUS**	**LIKELY PATHOGENIC**
Titin	lle22436del	c.67308_67310delAAT	**VUS**	**VUS**
MYH7	Glu374Val	g.23899001T>A	**VUS**	**VUS**
MYBPC3	Ala833Val	c.2498C>T	**VUS**	**BENIGN**
ANKRD1	Arg78Ser	c.234A>T	**VUS**	**LIKELY BENIGN**
MYBPC3	Trp1086Arg	c.3256T>C	**VUS**	**VUS**
MYBPC3 + FLNC	(Arg766Trp)	c.3815-10T>G c.2296C>T	**VUS BENIGN**	**VUS LIKELY BENIGN**
SOS1	Ser1096Thr	c.3286T>A	**VUS**	**LIKELY BENIGN**
TPM1	Asn202Ser	c.605A>G	**VUS**	**VUS**
RAF1	Met224Arg	c.671T>G	**VUS**	**VUS**
MYBPC3	Trp486Gly	c.1456T>G	**VUS**	**VUS**
TTN	His4271Gln	c.12813C>A	**VUS**	**VUS**
SOS1	Val624Phe	c.1870G>T	**VUS**	**VUS**
TNNI3	Pro82Ser	c.244C>T	**VUS**	**LIKELY BENIGN**
MYBPC3	Arg1228Cys	c.3682C>T	**VUS**	**LIKELY BENIGN**

Duplicate variants identified in more than one family removed.

Supplementary table 2: **Type of genetic testing performed in patients with a childhood diagnosis of hypertrophic cardiomyopathy by era.**

	Type of genetic testing in those with phenotype		Size of genetic panel in genetic index case			
Year genetic testing performed	Predictive testing	Panel testing	Sanger sequencing	Small NGS panel	Expanded NGS panel	Unknown size
**Pre 2000**	-	-	-	-	-	-
**2001-2005**	1	1	-	1	-	-
**2006-2010**	4	5	7	3	-	2
**2011-2015**	-	1	2	2	-	1
**2016 onwards**	13	10	-	7	9	-
**Unknown year of testing**	4	-	-	-	-	6
	***22 (39%)**	**17 (30%)**	**9 (22.5%)**	**13(32.5%)**	**9(22.5%)**	**9 (22.5%)**

**3 patients underwent predictive testing for a previously classified pathogenic variant, which was subsequently re-classified as a VUS*
